# Plantar pressures and ankle kinematics following anterior tibialis tendon transfers in children with clubfoot

**DOI:** 10.1186/1757-1146-5-S1-O32

**Published:** 2012-04-10

**Authors:** Kirsten Tulchin, Kelly A Jeans, Lori A Karol, Lindsey Crawford

**Affiliations:** 1 Texas Scottish Rite Hospital for Children, Dallas, Texas, 75219, USA; 2 John Peter Smith Hospital, Fort Worth, Texas, 76104, USA

## Background

Relapses following nonoperative treatment for clubfoot occur in 29-37% of feet following initial correction [1]. In patients with residual clubfoot deformity, excessive medial pull of the anterior tibialis muscle can lead to persistent supination and inversion of the forefoot [1]. The purpose of this study was to assess kinematic and plantar pressure changes following an ATT transfer.

## Materials and methods

Thirty children (37 feet) were evaluated pre- and 2.0±0.6 yrs (range: 0.8 to 2.9) post-op following ATT transfer. Foot progression angle (FPA) and sagittal ankle kinematics were assessed using a VICON system. Plantar pressures were collected using the Emed ST Platform. Representative trials were chosen for each subject for gait and plantar pressures. Plantar pressures were divided into medial and lateral hindfoot, midfoot and forefoot. Variables included: contact time (CT%), contact area (CA% total), peak pressure (PP), hindfoot-forefoot angle [2], deviation of the center-of-pressure (COP) line and region of initial contact. Twenty age matched controls were used for comparison.

## Results

Changes in plantar measures in the hindfoot show normalization of the CA% and CT% post-op (Table 1). The forefoot shows the most change with a significant decrease in CA%, CT% and PP in the lateral forefoot, redistributed to the first metatarsal for more even distribution through the foot. Initial contact was not different from normal post-op and no change was seen in the deviation of the COP line or hindfoot-forefoot angle (p=0.8025) post-op. Kinematically, patients with greater than 5° internal FPA had a higher likelihood of a successful outcome (60% had a less internal FPA, while no feet worsened.) Those that demonstrated a normal FPA pre-op risked a worsening FPA (35% had a more internal FPA while only 12% improved.) There were 16/37 (43%) feet with foot drop in late swing pre-op, 10 of which improved following surgery, however, 5 new foot drops developed post-op.

**Table 1:** Plantar pressures comparing Pre-Op, Post-Op & Control

	**Forefoot**				**Midfoot**				**Forefoot**					
	**Medial**		**Lateral**		**Medial**		**Lateral**		**1st Met.**		**2nd Met.**		**3-5th Mets.**	
	
	**Mean**	**+SD**	**Mean**	**+SD**	**Mean**	**+SD**	**Mean**	**+SD**	**Mean**	**+SD**	**Mean**	**+SD**	**Mean**	**+SD**
	
**Peak Pressure**	<0.0001 ‡*†		<0.0001 ‡*†		0.0001 ‡*		<0.0001 ‡*		<0.0001 ‡*		<0.0001 ‡*		<0.0001 ‡*	
Pre	10.2	6.6	10.6	4.8	4.4	2.6	16.2	6.3	4.4	2.6	9.1	3.7	23.8	10.3
Post	17.2	11.4	15.4	7.1	6.5	3.2	10.0	3.3	6.5	3.2	12.4	4.6	16.0	4.0
Control	25.0	8.2	20.8	6.4	7.5	2.0	7.9	1.8	7.5	2.0	14.7	4.9	14.5	4.7
**Contact Area**%	**0.0011 ‡**		0.3299		**0.0012 ‡**		<0.0001 ‡*†		<0.0001 ‡*†		0.5241		<0.0001 ‡*†	
Pre	9.1	3.4	13.2	4.8	2.1	2.1	24.8	2.9	2.1	2.1	8.4	2.3	26.6	5.2
Post	10.4	2.3	12.7	2.7	3.8	3.6	20.6	2.6	3.8	3.6	8.4	1.2	22.6	4.8
Control	11.8	1.1	11.7	1.0	5.2	3.4	16.0	2.7	5.2	3.4	8.9	1.5	17.8	2.5
**Contact Time**%	0.0011 ‡*		0.0408		0.0026 ‡*		**<0.0001 ‡†**		<0.0001 ‡*		0.0006 *		<0.0001 ‡*†	
Pre	38.8	21.9	48.2	18.9	31.6	22.7	80.3	8.0	31.6	22.7	75.7	15.1	94.5	3.5
Post	54.4	17.1	57.2	15.0	47.2	20.9	74.6	9.5	47.2	20.9	86.9	10.7	90.9	5.1
Control	50.7	9.0	49.9	8.5	45.2	9.2	61.8	10.8	45.2	9.2	82.0	6.3	83.9	5.0

Significant change (p< 0.05): ‡Pre/Control; †Post/Control; *Pre/Post

## Conclusions

Changes seen in plantar pressures would suggest that during stance, the foot is better aligned, more evenly distributing pressures throughout the foot rather than focused to the lateral midfoot and forefoot regions. Based on gait results, pre-operative foot progression angle may be an indicator for successful outcomes of ATT transfer in patients with residual deformity.
